# DT-SCNN: dual-threshold spiking convolutional neural network with fewer operations and memory access for edge applications

**DOI:** 10.3389/fncom.2024.1418115

**Published:** 2024-05-30

**Authors:** Fuming Lei, Xu Yang, Jian Liu, Runjiang Dou, Nanjian Wu

**Affiliations:** ^1^State Key Laboratory of Superlattices and Microstructures, Institute of Semiconductors, Chinese Academy of Sciences, Beijing, China; ^2^Center of Materials Science and Optoelectronics Engineering, University of Chinese Academy of Sciences, Beijing, China

**Keywords:** spiking neural network, dual-threshold, network compression, edge application, backpropagation

## Abstract

The spiking convolutional neural network (SCNN) is a kind of spiking neural network (SNN) with high accuracy for visual tasks and power efficiency on neuromorphic hardware, which is attractive for edge applications. However, it is challenging to implement SCNNs on resource-constrained edge devices because of the large number of convolutional operations and membrane potential (Vm) storage needed. Previous works have focused on timestep reduction, network pruning, and network quantization to realize SCNN implementation on edge devices. However, they overlooked similarities between spiking feature maps (SFmaps), which contain significant redundancy and cause unnecessary computation and storage. This work proposes a dual-threshold spiking convolutional neural network (DT-SCNN) to decrease the number of operations and memory access by utilizing similarities between SFmaps. The DT-SCNN employs dual firing thresholds to derive two similar SFmaps from one Vm map, reducing the number of convolutional operations and decreasing the volume of Vms and convolutional weights by half. We propose a variant spatio-temporal back propagation (STBP) training method with a two-stage strategy to train DT-SCNNs to decrease the inference timestep to 1. The experimental results show that the dual-thresholds mechanism achieves a 50% reduction in operations and data storage for the convolutional layers compared to conventional SCNNs while achieving not more than a 0.4% accuracy loss on the CIFAR10, MNIST, and Fashion MNIST datasets. Due to the lightweight network and single timestep inference, the DT-SCNN has the least number of operations compared to previous works, paving the way for low-latency and power-efficient edge applications.

## 1 Introduction

Spiking neural networks (SNNs) are inspired by the brain and use spikes (binary signals) to transmit information between neurons. Neuromorphic hardware only requires processing the spike-based accumulate (ACC) operations, effectively bypassing the need to compute zero input values to attain remarkable power efficiency. Consequently, SNNs exhibit significant energy efficiency when implemented on neuromorphic hardware (Pei et al., 2019), making them increasingly appealing for edge applications (Zhang et al., 2020; Liu and Zhang, 2022). Spiking convolutional neural networks (SCNNs) is a kind of SNN widely used in vision tasks (Cao et al., 2015; Kheradpisheh et al., 2018) with accuracy similar to convolutional neural networks (Wu et al., 2019). It consists of convolutional, pooling, and fully connected layers. The SCNNs extract image features through hierarchical convolutional layers, providing strong image processing capabilities. Each convolutional layer generates many spiking feature maps (SFmaps) from the same number of membrane potentials (Vm). As a kind of SNN, SCNNs also have high energy efficiency in neuromorphic hardware. However, SCNNs must generate several SFmaps to ensure a high processing accuracy, leading to many convolution operations, weights, and Vm storage. This makes deploying SCNNs on edge devices difficult due to the limited computing power, power consumption, and storage capacity. Researchers have made significant efforts to solve this issue. First, some methods are proposed to decrease the timesteps^1^ to decrease operations and memory access. SCNNs have achieved high precision with few timesteps (Chowdhury et al., 2021), with the spatio-temporal backpropagation (STBP) training method (Zhu and Shi, 2018), direct input encoding (Wu et al., 2019), and re-training strategy (Chowdhury et al., 2021). Second, a series of methods have been proposed to compact SCNNs, such as network pruning (Liu et al., 2022; Schaefer et al., 2023) to increase the sparsity and low-bit quantization (Kheradpisheh et al., 2022; Shymyrbay et al., 2022) to reduce the computational precision.

**Figure 1:**
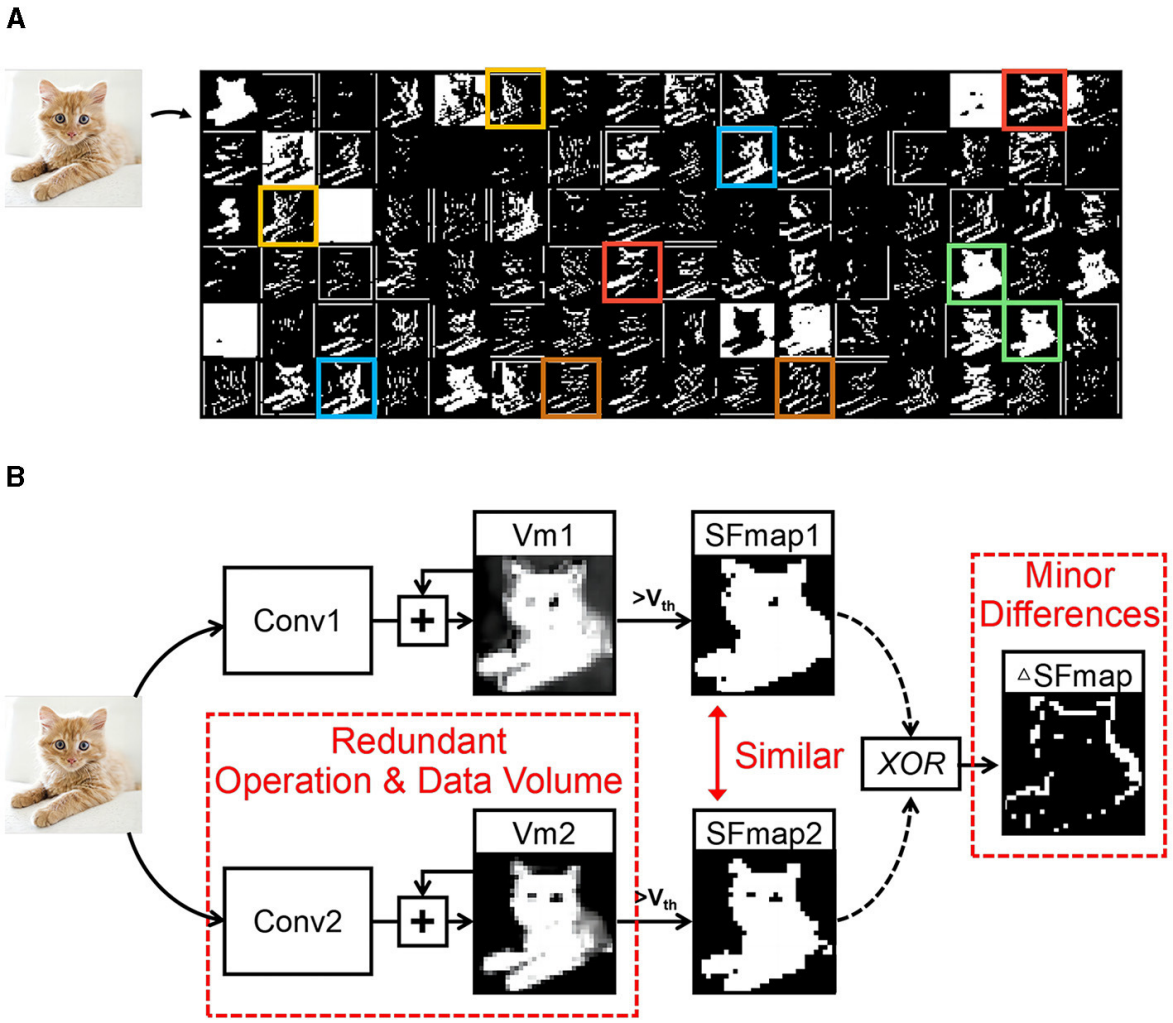
Similarity between SFmaps and their generation process. **(A)** The SFmaps of a SCNN convolutional layer where those corresponding to the same color boxes have great similarity and **(B)** generation process of two similar SFmaps.

However, these studies overlook the similarity between SFmaps, leading to wasteful calculations. Figure 1A displays the SFmaps of the 1st convolutional layer of a typical SCNN. Pairs of similar SFmaps are marked with boxes of the same color. Figure 1B shows the generation process of two similar SFmaps. The input maps are processed through convolution operations to update two Vm maps. Each Vm map generates an SFmap via threshold comparisons. There are minor differences (ΔSFmaps) between two similar SFmaps, but they are obtained through the processes described above, resulting in redundant operations and data volume. The study (Han et al., 2020) indicates that similarity in feature maps is vital for achieving high accuracy. Therefore, there are challenges to reduce these redundancies while preserving similar SFmaps.

To address this challenge, this work proposes a novel lightweight dual-threshold spiking convolutional neural network (DT-SCNN) model and a variant spatio-temporal back propagation (STBP) training method. We simplify the training process in Chowdhury et al. (2021) into a two-stage training strategy to train DT-SCNNs with only one timestep. The DT-SCNN uses dual-threshold to obtain two similar spike feature maps from one Vm map, reducing the number of convolutional weights and Vm values by half with minimal impact on the accuracy. As the network model is lightweight and requires only a single timestep, the number of operations and memory access can be significantly reduced, paving the way for low-latency and power-efficient edge visual applications.

This work proceeds as follows. Section 2 briefly reviews the general concept of conventional SCNNs and proposes the DT-SCNN model. The training implementation of the DT-SCNN is also introduced. Section 3 analyzes the experimental performance and compares it to other works. Finally, Section 4 discusses and concludes this work.

## 2 Methods

This section first describes the general concept and shortcomings of conventional SCNNs. We then present the proposed DT-SCNN and analyze its characteristics, such as fewer operations and memory access. Finally, we introduce the variant STBP training method with a two-stage strategy for DT-SCNNs.

### 2.1 Overview and redundancy of normal SCNN

The SCNN consists of alternately arranged convolutional layers and pooling layers, and fully connected layers. Each convolutional layer extracts features into output SFmaps. The pooling layer combines the neuron cluster outputs from one SFmap into the input of one neuron in the next layer. The first convolutional layer of the SCNN acts as a coding layer to directly process the real-valued picture (Wu et al., 2019) and generate SFmaps. The coding layer has the same neuron dynamics as other layers. The final SCNN layer is the classifier, taking the Vms at the last timestep as the network output. For classification tasks, the neuron label with the largest Vm in the output layer represents the predicted category.

**Figure 2:**
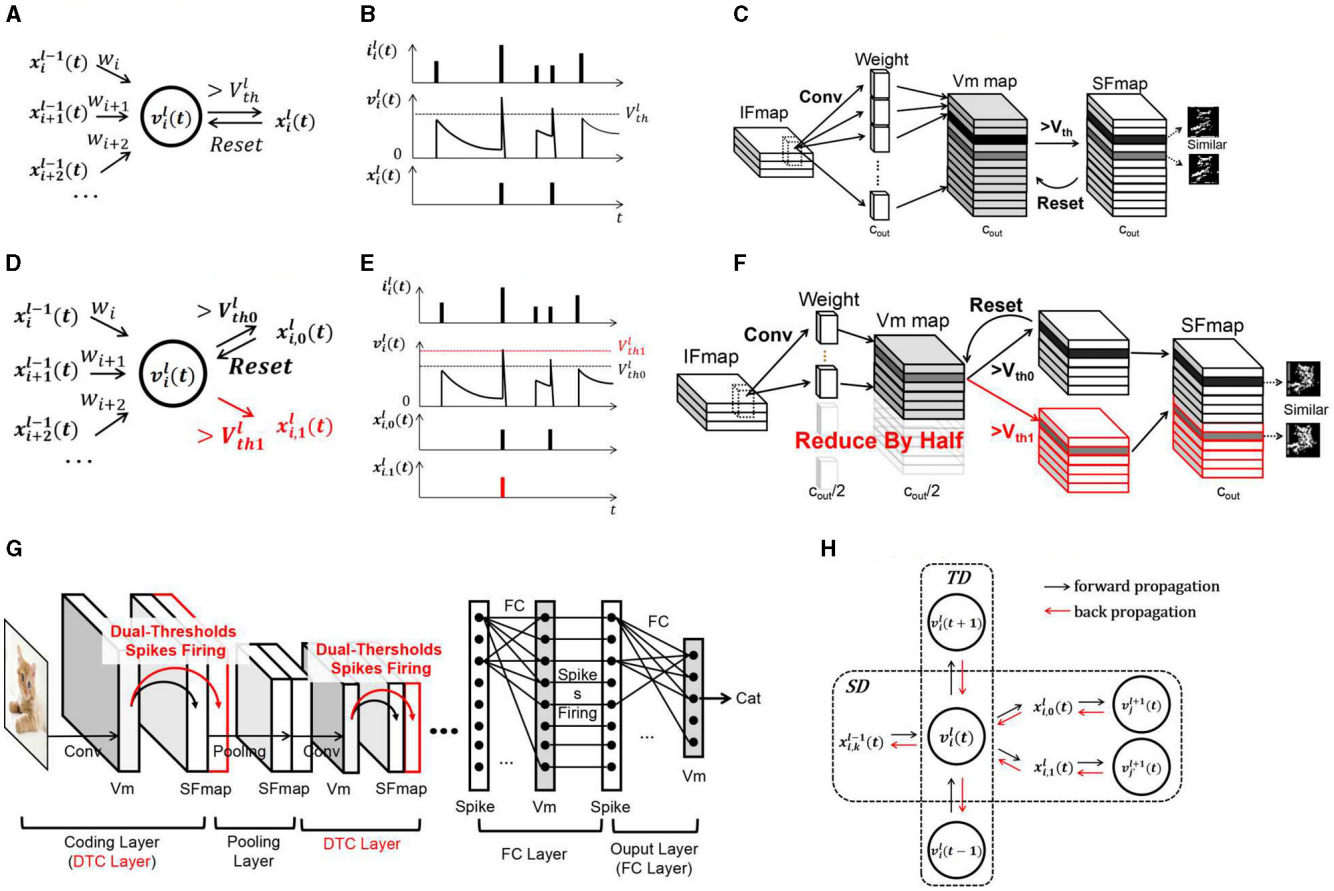
Diagram of the SCNN and DT-SCNN. **(A)** Schematic diagram of LIF neurons, **(B)** update of LIF neurons, **(C)** convolutional layer in the SCNN, **(D)** schematic diagram of DT-LIF neurons, **(E)** update of the DT-LIF Vm, **(F)** dual-threshold spiking convolutional layer in the DT-SCNN, **(G)** basic structure of the DT-SCNN, and **(H)** error propagation in the STD of the DT-LIF.

The leaky integrate and fire (LIF) neuron is a kind of neuronal model usually adopted for SCNNs because of its simplicity and hardware friendliness. This kind of neuron is described in Figures 2A, B. The Vm is governed by (Equations 1, 2):


(1) $$\begin{array}{l} v_{i}^{l}\left(t\right)=\lambda v_{i}^{l}\left(t-1\right)\left(1-x_{i}^{l}\left(t-1\right)\right)+i_{i}^{l}\left(t\right) \end{array}$$

(2) $$x_{i}^{l}(t)=\{\begin{array}{ll} 1, & v_{i}^{l}(t)\geq V_{th}^{l}\\ 0, & otherwise \end{array}$$

where $v_{i}^{l}\left(t\right)$, $x_{i}^{l}\left(t\right)$, and $i_{i}^{l}\left(t\right)$ are the Vm, output, and synaptic current of neuron *i* in the *l*-layer at time *t*, respectively. The λ is the leakage factor. When the Vm reaches the threshold *V*_*th*_, the spike is fired and the Vm resets to 0. The synaptic current $i_{i}^{l}\left(t\right)$ is given by:


(3) $$\begin{array}{l} i_{i}^{l}\left(t\right)=\underset{j}{\sum }w_{j}^{l}x_{j}^{l-1}\left(t\right) \end{array}$$

where $w_{j}^{l}$ is the synaptic weight connected to the *jth* neuron in layer *l*−1. In the coding layer, $x_{j}^{0}\left(t\right)$ is the input of the whole network, which is the gray-scale picture pixel value. Equation (3) requires multiply-accumulation (MAC) operations. In other layers, $x_{j}^{l}\left(t\right)$ is the spike from the pre-synaptic neuron, which is a binary value. As shown in Equation (3), only ACC operations are needed.

The operations within SCNNs are concentrated primarily in the convolutional layers. Figure 2C illustrates a convolutional layer. The input feature maps (IFmaps) of size (*h, w, c*_*in*_) are processed through convolution operations to update the Vm maps of size $\left(h^{\prime },w^{\prime },c_{out}\right)$. The Vm maps generate SFmaps of the same size $\left(h^{\prime },w^{\prime },c_{out}\right)$. The number of MAC or ACC operations in a convolutional layer is $h^{\prime }\cdot w^{\prime }\cdot c_{in}\cdot k\cdot k\cdot c_{out}$. To obtain rich features, the number of Vm maps, SFmaps, and weight kernels *C*_*out*_ are always significant, such as 128 and 256, giving high operations and memory access. As analyzed in the introduction, there is also considerable redundancy.

### 2.2 Proposed dual-threshold SCNN and dual-threshold LIF model

Information transmitted between layers in the SCNN is encoded as 1-bit spikes generated from multi-bit Vms. The information capacity of Vms is much larger than that of SFmaps, meaning that the information of two similar SFmaps may be contained in one Vm map. Therefore, this work proposes the DT-SCNN model with dual-threshold LIF (DT-LIF) neurons to generate two SFmaps from one Vm map using two thresholds, as shown in Figure 2G. The DT-LIF neuron model is shown in Figures 2D, E defined as (Equation 4):


(4) $$\begin{array}{l} v_{i}^{l}\left(t\right)=\lambda v_{i}^{l}\left(t-1\right)\left(1-x_{i,0}^{l}\left(t-1\right)\right)+i_{i}^{l}\left(t\right) \end{array}$$

(5) $$x_{i,0}^{l}(t)=\{\begin{array}{ll} 1, & v_{i}^{l}(t)\geq V_{th0}^{l}\\ 0, & otherwise \end{array}$$

(6) $$x_{i,1}^{l}(t)=\{\begin{array}{ll} 1, & v_{i}^{l}(t)\geq V_{th1}^{l}\\ 0, & otherwise \end{array}$$

where a Vm $v_{i}^{l}\left(t\right)$ generates two spikes $x_{i,0}^{l}\left(t\right)$ and $x_{i,1}^{l}\left(t\right)$ from two different thresholds *V*_*th*0_ and *V*_*th*1_. The Vm is reset only when it exceeds *V*_*th*0_, and the values of two thresholds are determined via training. The DT-LIF neuron model neither introduces complex operations nor increases hardware complexity when deployed on edge devices.

The convolutional layers composed of DT-LIF neurons are called dual-threshold convolutional (DTC) layers, as shown in Figure 2F. Two similar SFmaps are generated by one Vm map. The SFmaps generated by two thresholds are concatenated in the channel dimension as (Equation 7):


(7) $$\begin{array}{l} X^{l}\left(t\right)=cat\left(X_{0}^{l}\left(t\right),X_{1}^{l}\left(t\right)\right) \end{array}$$

where $X_{0}^{l}\left(t\right)$ and $X_{1}^{l}\left(t\right)$ are the SFmaps generated by two thresholds, *X*^*l*^(*t*) is a tensor containing all SFmaps of the DTC layer, where $x_{i,0}^{l}\left(t\right)\in X_{0}^{l}\left(t\right)$, $x_{i,1}^{l}\left(t\right)\in X_{1}^{l}\left(t\right)$. To obtain SFmaps with the same size of $\left(h^{\prime },w^{\prime },c_{out}\right)$ as the SCNNs, the numbers of Vms and synaptic weights are reduced by half (*c*_*out*_/2), and their sizes are $\left(h^{\prime },w^{\prime },c_{out}/2\right)$ and (*c, k, k, c*_*out*_/2), respectively. The number of MAC or ACC operations for the convolutions in Equation (3) is also reduced by half, which is $h^{\prime }\cdot w^{\prime }\cdot c_{in}\cdot k\cdot k\cdot c_{out}/2$. Therefore, only half of the weights, Vms, and convolution operations are needed to obtain the same number of SFmaps as SCNNs.

The DT-SCNNs can be obtained by replacing convolutional layers in the SCNN with DTC layers. The basic structure is shown in Figure 2G. The SCNN's operations are primarily from convolutions. Thus, the operations of entire networks can be reduced by half. Fewer weights and Vms also reduce the demand for memory access. Although similar SFmaps are derived from the same Vms, they play different roles in feature extraction. The higher threshold is used to extract the critical features, while the lower threshold is employed to preserve the details. Combining critical and detailed information enables the subsequent layers to extract features more comprehensively, ensuring high accuracy.

### 2.3 Training implementation

This section introduces the training method for the DT-SNN. The DT-SCNN is trained based on the variant STBP with a two-stage strategy. The variant STBP is based on the STBP (Zhu and Shi, 2018). So in this work, we only present the main modifications in the gradient propagation path through the DTC layer. After forward propagation, the final output of the SCNN and object labels are used to calculate the loss function as *Loss*. The error is back propagated for adjusting the weights to minimize the *Loss*. As shown in Figure 2H, the information forward propagates in the DT-LIF neuron model through the layer-by-layer spatial domain (SD) and the temporal domain (TD). Therefore, the error backpropagation must pass through the spatial and temporal domain (STD). When calculating the derivative of *Loss* with respect to *x* and *v* in layer *l* at time *t*, the STBP backpropagates the gradients $\frac{\partial Loss}{\partial x_{i,0}^{l+1}\left(t\right)}+\frac{\partial Loss}{\partial x_{i,1}^{l+1}\left(t\right)}$ from neurons in layer *l*+1 and $\frac{\partial Loss}{\partial x_{i,k}^{l}\left(t+1\right)}$ from time *t*+1 as follows (Equations 8, 9):


(8) $$\begin{array}{c} \frac{\partial Loss}{\partial x_{i,k}^{l}(t)}=\sum_{j=1}^{N_{l+1}}\left(\frac{\partial Loss}{\partial x_{j,0}^{l+1}(t)}\frac{\partial x_{j,0}^{l+1}(t)}{\partial x_{i,k}^{l}(t)}+\frac{\partial Loss}{\partial x_{j,1}^{l+1}(t)}\frac{\partial x_{j,1}^{l+1}(t)}{\partial x_{i,k}^{l}(t)}\right)\\ +\frac{\partial Loss}{\partial x_{i,k}^{l}(t+1)}\frac{\partial x_{i,k}^{l}(t+1)}{\partial x_{i,k}^{l}(t)} \end{array}$$

(9) $$\begin{array}{l} \frac{\partial Loss}{\partial v_{i}^{l}(t)}=(\frac{\partial Loss}{\partial x_{i,0}^{l}(t)}\frac{\partial x_{i,0}^{l}(t)}{\partial v_{i}^{l}(t)}+\frac{\partial Loss}{\partial x_{i,1}^{l}(t)}\frac{\partial x_{i,1}^{l}(t)}{\partial v_{i}^{l}(t)})+\\ \;(\frac{\partial Loss}{\partial x_{i,0}^{l}(t+1)}\frac{\partial x_{i,0}^{l}(t+1)}{\partial v_{i}^{l}(t)}+\frac{\partial Loss}{\partial x_{i,1}^{l}(t+1)}\frac{\partial x_{i,1}^{l}(t+1)}{\partial v_{i}^{l}(t)}) \end{array}$$

where *k* ∈ {0, 1} represents the two threshold pathways, and *N*_1+1_ represents the number of neurons in layer *l*+1 connected to this neuron. The gradients from two threshold pathways converge to one Vm. Therefore, the weights can learn the method to encode information of two SFmaps into a single Vm map. Based on STBP, the gradients of two thresholds can be computed by (Equation 10):


(10) $$\begin{array}{l} \Delta V_{th,k}^{l}=-\gamma \frac{\partial Loss}{\partial V_{th,k}^{l}} \end{array}$$

where, γ is learing rate. Combining Equations (5, 6, 11):


(11) $$\begin{array}{l} \frac{\partial Loss}{\partial V_{th,k}^{l}}=\sum_{i}\frac{\partial Loss}{\partial x_{i,k}^{l}(t)}\frac{\partial x_{i,k}^{l}(t)}{\partial V_{th,k}^{l}}\\ \;=\sum_{i}\frac{\partial Loss}{\partial x_{i,k}^{l}(t)}\frac{\partial x_{i,k}^{l}(t)}{\partial (v_{i}^{l}(t)/V_{th,k}^{l})}\frac{\partial (v_{i}^{l}(t)/V_{th,k}^{l})}{\partial V_{th,k}^{l}} \end{array}$$

To avoid overly complex gradient chains, we ignored the differentiation of $v_{i}^{l}\left(t\right)$ and $V_{th,k}^{l}$ (Equation 12):


(12) $$\begin{array}{l} \frac{\partial \left(v_{i}^{l}\left(t\right)/V_{th,k}^{l}\right)}{\partial V_{th,k}^{l}}=-\frac{v_{i}^{l}\left(t\right)}{{\left(V_{th,k}^{l}\right)}^{2}} \end{array}$$

Due to the non-differentiability of the spikes firing process in Equations (5, 6), using surrogate gradients *h*(*u*) to approximate its differentiation (Zhu and Shi, 2018) (Equation 13, 14):


(13) $$\begin{array}{l} \frac{\partial x_{i,k}^{l}\left(t\right)}{\partial \left(v_{i}^{l}\left(t\right)/V_{th,k}^{l}\right)}=h\left(u\right),\;u=v_{i}^{l}\left(t\right)/V_{th,k}^{l} \end{array}$$

(14) $$\begin{array}{l} h\left(u\right)=sign\left(|u-1|<\frac{1}{2}\right) \end{array}$$

We use a two-stage re-training strategy to reduce the timestep to 1 based on the variant STBP training method. The method of Chowdhury et al. (2021) gradually reduces the number of timesteps to avoid gradient disappearance when directly training timesteps to 1. Therein, we divided the training processing into two stages of pre-train and re-train. The training starts with random weights with five timesteps in the pre-train stage. The re-training progress starts from the pre-trained model and sets the timestep to one in the re-train stage. The networks with one timestep still maintain a high accuracy. In subsequent experiments, we also used the two-stage re-training strategy based on the STBP to train conventional SCNNs.

## 3 Experiments and results

### 3.1 Datasets and settings

We tested the proposed model on three image datasets MNIST (Lecun et al., 1998), FashionMNIST (Xiao et al., 2017), and CIFAR10 (Krizhevsky and Hinton, 2009). To ensure a fair comparison with several previously state-of-the-art results, we designed the networks to have the same or similar size. As we focused on edge applications, we limited the network weights to a few megabytes (Luo et al., 2022; Wang et al., 2022).

Table 1 illustrates the three networks developed for comparison: SCNN baseline, DT-SCNN, and HC-SCNN. The DT-SCNN replaces the spiking convolutional layers in the SCNN baseline with the DTC layers while maintaining the same number of SFmaps. To determine the impact of reduced SFmaps on the accuracy, the HC-SCNN is an SCNN with half the number of SFmaps compared to the SCNN baseline. The HC-SCNN and DT-SCNN have the same number of Vms.

**Table 1:** Comparison of the proposed approach with the conventional SCNN in different datasets.

**Name**	**Model**	**Network**	**Accuracy**	**Operations** ^*1^	**Weights** ^*2^	**Vms** ^*3^
Net1 (CIFAR10)	SCNN Baseline	96c3-256c3-p2-384c3-p2-384c3-256c3-1024fc-1024fc-10fc	89.65%	612M(ACC) +2.65M(MAC)	3.32M	0.5M
	**DT-SCNN**	**48dtc** ^*^ **3-128dtc3-p2-192dtc3-p2-192dtc3-128dtc3-p2-1024fc-10fc**	**89.31% (↓0.34%)**	**315M(ACC) +1.33M(MAC) (↓49%)**	**1.66M (↓50%)**	**0.25M (↓50%)**
	HC-SCNN	48c3-128c3-p2-192c3-p2-192c3-128c3-1024fc-1024fc-10fc	88.39% (↓1.26%)	158M(ACC) +1.33M(MAC) (↓74%)	0.83M (↓75%)	0.25M (↓50%)
Net2 (MNIST)	SCNN Baseline	16c5-p2-40c5-p2-256fc-10fc	99.43%	3.6M(ACC) +0.31M(MAC)	16.4K	20.7K
	**DT-SCNN**	**8dtc5-p2-20dtc5-p2-256fc-10fc**	**99.37% (↓0.06%)**	**2.1M(ACC) +0.16M(MAC) (↓45%)**	**8.2K (↓50%)**	**10.5K (↓50%)**
	HC-SCNN	8c5-p2-20c5-p2-256fc-10fc	99.21% (↓0.22%)	1.0M(ACC) +0.16M(MAC) (↓70%)	4.2K (↓74%)	10.5K (↓50%)
Net3 (Fashion MNIST)	SCNN Baseline	16c5-p2-32c5-p2-256fc-10fc	92.46%	2.9M(ACC) +0.31(MAC)	13.2K	19.1K
	**DT-SCNN**	**8dtc5-p2-16dtc5-p2-256fc-10fc**	**92.25% (↓0.21%)**	**1.7M(ACC) +0.16(MAC) (↓46%)**	**6.6K (↓50%)**	**9.7K (↓50%)**
	HC-SCNN	8c5-p2-16c5-p2-256fc-10fc	91.34% (↓1.12%)	0.83M(ACC) +0.16(MAC) (↓69%)	3.4K (↓74%)	9.7K (↓50%)

^*^α*dtcβ* means double-threshold convolution, α is the number of convolution kernels and Vm maps, 2α is the number of SFmaps obtained after double-threshold firing, and β is the size of the convolution kernels.

^*1^ Number of ACC and MAC operations across the network.

^*2^ Number of weights in the convolutional or the dual-threshold convolutional layers.

^*3^ Number of membrane potential values (Vms) across the entire network. The experimental results of DT-SCNNs proposed in this work are highlighted in bold.

The networks were trained using the two-stage re-training strategy. For Net1, each stage trained for 400 rounds, and the network learning rate dropped by 10% every 100 rounds. For Net2 and Net3, each stage trained for 100 rounds, and the network learning rate dropped by 10% every 25 rounds. All thresholds were initialized to the same value of 0.5. Dropout was used to avoid overfitting with a probability set to 0.5, and the leakage factor λ was 0.5. The CIFAR10 dataset was normalized, and random cropping and mirroring were applied for data augmentation. Our code will be made publicly available at: https://github.com/flaviomarix/DT-SCNN/.

### 3.2 Accuracy and computing cost of normal SCNN and DT-SCNN

Table 1 shows each network's recognition accuracy, computing cost, and latency under the three datasets. Compared to the SCNN baseline, the DT-SCNN exhibits a minimal accuracy reduction, with decreases of only 0.34, 0.06, and 0.21% on the CIFAR10, MNIST, and Fashion MNIST datasets, respectively. In contrast, the HC-SCNN led to a significant accuracy drop, indicating that simply reducing the number of SFmaps does not retain the accuracy.

In terms of computational cost, the DT-SCNN reduced the number of operations by nearly 50% over the SCNN baseline. More operations are removed for networks with a higher proportion of convolution layers. Such as Net1-DT-SCNN achieved a 49% operations reduction. In addition, the number of convolutional weights and Vms were reduced by half, resulting in less memory access. Although the HC-SCNN has fewer operations, weights, and Vms, the excessive accuracy loss is unacceptable. The accuracy loss of HC-SCNN is about four times of the accuracy loss of DT-SCNN. In short, the DT-SCNN significantly reduces the operations and memory access requirements while maintaining nearly the same accuracy. The DT-SCNN has less latency and power consumption in edge applications thanks to fewer operations and memory accesses.

### 3.3 Dual threshold and visualization of feature maps

**Figure 3:**
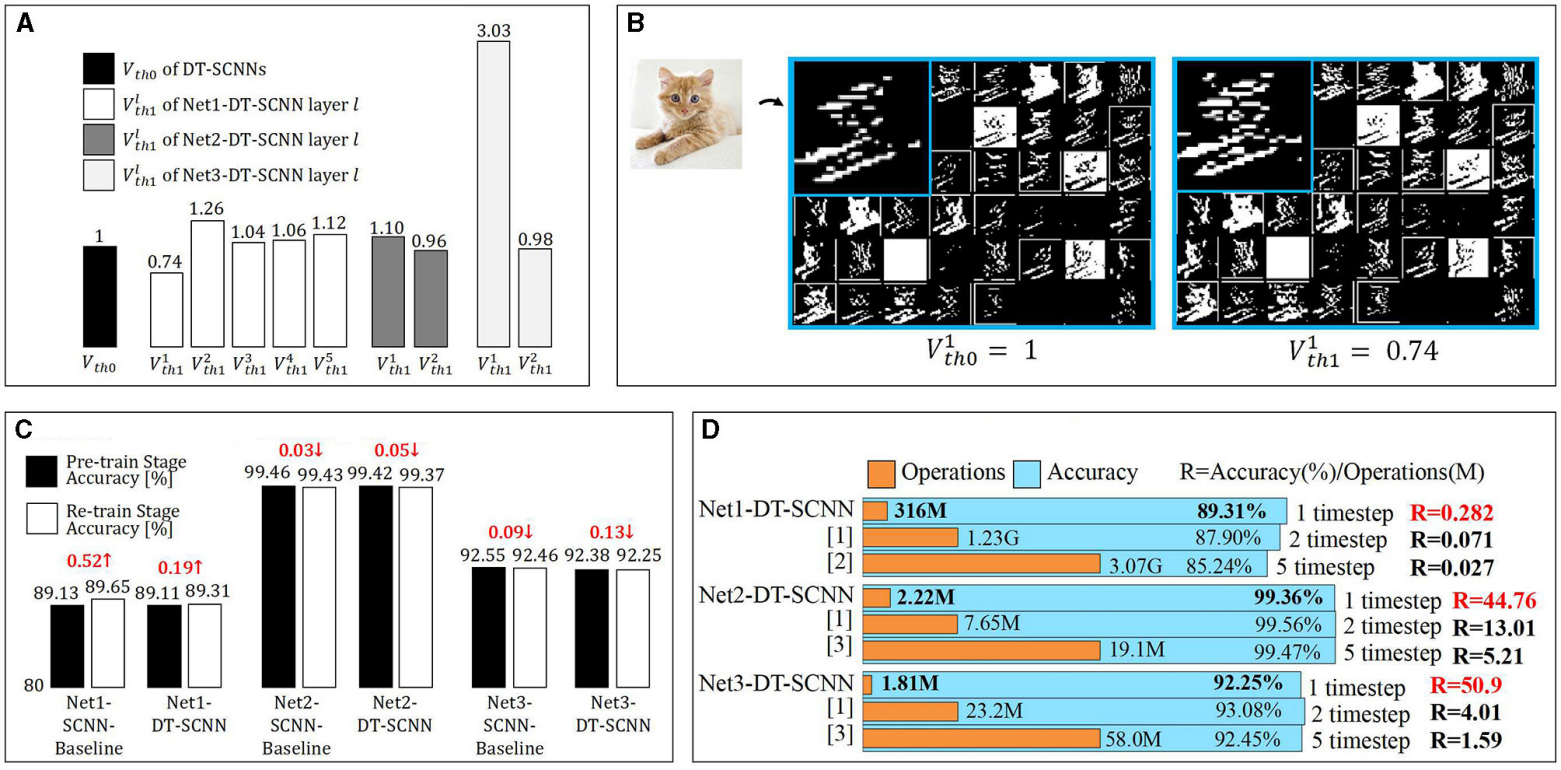
**(A)** Dual-thresholds for each DTC layer in DT-SCNNs and **(B)** visualizations of SFmaps generated by the first DTC layer in Net1-DT-SCNN. The left and right SFmaps are fired by $V_{th0}^{1}$ and $V_{th1}^{1}$, respectively. **(C)** Accuracy of the two-stage re-training strategy and **(D)** comparison of the state-of-the-art with the proposed work in terms of operations and accuracy. The number of operations for a single inference of the entire network is multiplied by the number of timesteps. [1] (Xu et al., 2022), [2] (Wu et al., 2019), and [3] (Zhang and Li, 2020).

The dual thresholds of each layer in the DT-SCNN are given in Figure 3A. For demonstration purposes, we scale the two thresholds of each layer equally to make *V*_*th*0_ = 1. The *V*_*th*0_ and *V*_*th*1_ are initialized to the same value but differ after training. The SFmaps generated by the dual thresholds are illustrated in Figure 3B for the first layer of the Net1-DT-SCNN. The SFmaps in the left and right are fired by *V*_*th*0_ and *V*_*th*1_, respectively. Although each SFmap pair is fired from the same Vm map, there are still some differences, meaning that sufficiently rich SFmaps can be generated from halved Vms. The SFmaps for the high threshold filter out more critical information, while the SFmaps of the low threshold preserve more details. This ensures the accuracy of the DT-SCNNs.

### 3.4 Two-stage re-training strategy accuracy

We use the two-stage re-training strategy to train the SCNNs and DT-SCNNs. Figure 3C shows the network's accuracy and differences in the two stages. Despite having more timesteps in the pre-train stage (five timesteps) than in the re-train stage (one timestep), the accuracy loss in the re-train stage relative to the pre-train stage is < 0.13% and is even improved for Net1. This result shows that the two-stage re-training method is effective at reducing the timestep to 1 while maintaining a high accuracy.

### 3.5 Comparison with state-of-the-art results

We compared DT-SCNN with several previously reported state-of-the-art results obtained using similar network sizes, as shown in Figure 3D. The accuracy of the proposed DT-SCNN is similar to or exceeds previous works with far fewer operations thanks to DTC layers and single timestep. We define R = Accuracy (%)/num.Operations(M) to qualitatively analyze the relationship between the number of operations and accuracy in different networks. The R of DT-SCNN is significantly higher than that of other works. It means that the DT-SCNN achieves higher accuracy with fewer operations, which can prove that the DT-SCNN is the best choice after balancing efficiency and accuracy.

## 4 Discussion

This work proposed a lightweight DT-SCNN structure that reduces the number of operations and memory access of SCNNs with minimal accuracy loss. A variant STBP training method with a two-stage strategy reduces the timestep of the DT-SCNNs to 1 to reduce the computing delay and power consumption of SCNNs when deployed on edge devices. Experimental analyzes show that the DT-SCNNs are the best choice for balancing trade-offs between the computational requirements and accuracy for edge applications. This work only investigated the effect of applying two thresholds to convolutional layers. Future work will explore more thresholds or apply multiple thresholds to different types of layers, and expand DT-SCNN to larger networks.

## Data availability statement

The original contributions presented in the study are included in the article/supplementary material, further inquiries can be directed to the corresponding author.

## Author contributions

FL: Writing—review & editing, Writing—original draft, Visualization, Validation, Supervision, Software, Resources, Project administration, Methodology, Investigation, Formal analysis, Data curation, Conceptualization. XY: Writing—review & editing. JL: Writing—review & editing. RD: Writing—review & editing. NW: Writing—review & editing.

## Footnotes

^1^Timestep is the unit of time taken by each input frame to be processed through all layers of the model.
